# Cranial biomechanics in basal urodeles: the Siberian salamander (*Salamandrella keyserlingii*) and its evolutionary and developmental implications

**DOI:** 10.1038/s41598-017-10553-1

**Published:** 2017-08-31

**Authors:** Zupeng Zhou, Josep Fortuny, Jordi Marcé-Nogué, Pavel P. Skutschas

**Affiliations:** 1 School of Mechanical and Electrical Engineering, Guilin University of Electronic Technology, Guilin, China; 2 Centre de Recherches en Paléobiodiversité et Paléoenvironnements, Muséum National d’Histoire Naturelle, Bâtiment de Paléontologie, CP38, 8 rue Buffon, 75005 Paris, France; 3 Institut Català de Paleontologia M. Crusafont. Z building, Universitat Autònoma de Barcelona, CP:, 08193 Cerdanyola del Vallès Barcelona, Spain; 4 Centrum für Naturkunde, University of Hamburg, CP:, 20146 Hamburg, Germany; 5 Faculty of Biology, Department of Vertebrate Zoology, Saint Petersburg State University, Saint Petersburg, Russia

## Abstract

Developmental changes in salamander skulls, before and after metamorphosis, affect the feeding capabilities of these animals. How changes in cranial morphology and tissue properties affect the function of the skull are key to decipher the early evolutionary history of the crown-group of salamanders. Here, 3D cranial biomechanics of the adult *Salamandrella keyserlingii* were analyzed under different tissue properties and ossification sequences of the cranial skeleton. This helped unravel that: (a) Mechanical properties of tissues (as bone, cartilage or connective tissue) imply a consensus between the stiffness required to perform a function versus the fixation (and displacement) required with the surrounding skeletal elements. (b) Changes on the ossification pattern, producing fontanelles as a result of bone loss or failure to ossify, represent a trend toward simplification potentially helping to distribute stress through the skull, but may also imply a major destabilization of the skull. (c) Bone loss may be originated due to biomechanical optimization and potential reduction of developmental costs. (d) Hynobiids are excellent models for biomechanical reconstruction of extinct early urodeles.

## Introduction

Skeletal development of urodeles (crown-group salamanders) is mainly influenced by their complex life history: developing morphologically and ecologically distinctive larval and postmetamorphic stages, and undergoing metamorphosis, neoteny or direct development as different developing modes^1–3^. Ossification patterns and sequences of salamanders have been studied in different groups, and reveal (a) the position and morphology of bone primordia and growth lines, (b) the morphological changes and chronological sequences, and (c) their evolutionary implications^2, 4–6^ (and references therein). These developmental changes have several functional – adaptative implications. Morphological changes in the skeletal structures (such as cartilage or bone elements, muscle developments and insertion positions, etc.) derive from this developmental changes^3, 7^. Of particular interest, developmental changes found in the salamander skull affect the feeding capability before and after metamorphosis. Generally, larvae are aquatic suction feeders while, in some groups, postmetamorphic juveniles and adults may continue as suction feeders or modify to perform jaw prehension and/or tongue protrusion^8, 9^. How these feeding strategies evolved, and their functional and morphological implications across the fish-tetrapod transition and the lissamphibian origin (particularly salamanders), have been extensively discussed using neo- and paleontological data, providing clues to decipher the evolutionary history of early tetrapods and particularly, urodeles^10–16^. Regarding extant urodeles, basal groups are of special interest to understand cranial morphological changes and their functional implications during the early history of the crown-group salamanders, and how of these changes and implications relate to the development of the cranial structures. Therefore, cryptobranchids have been particularly studied in their osteology, myology and functional morphology, due to their basal phylogenetic position within Urodela, gigantic size and the capability to perform unique asymmetrical modes of feeding^12, 14, 17, 18^ (and references therein). However, little information is known about the other basal group of urodeles, Hynobiidae. For this reason we analyze the feeding biomechanics of the basal salamander group Hynobiidae. Hynobiids and cryptobranchids are united in the suborder Cryptobranchoidea as a basal clade of urodeles^7, 19, 20^. The family Hynobiidae includes about nine to eleven genera, 66*–*67 species of small-medium sized members (10*–*25 cm total length) of terrestrial – semi-aquatic salamanders^21, 22^ that use jaw prehension for feeding, both on land and in water (e.g. *Salamandrella keyserlingii* and *Paradactylodon persicus* ^9, 23^). Hynobiids retain countable primitive characters in their cranial morphology and development^1, 4, 24^.

**Figure 1:**
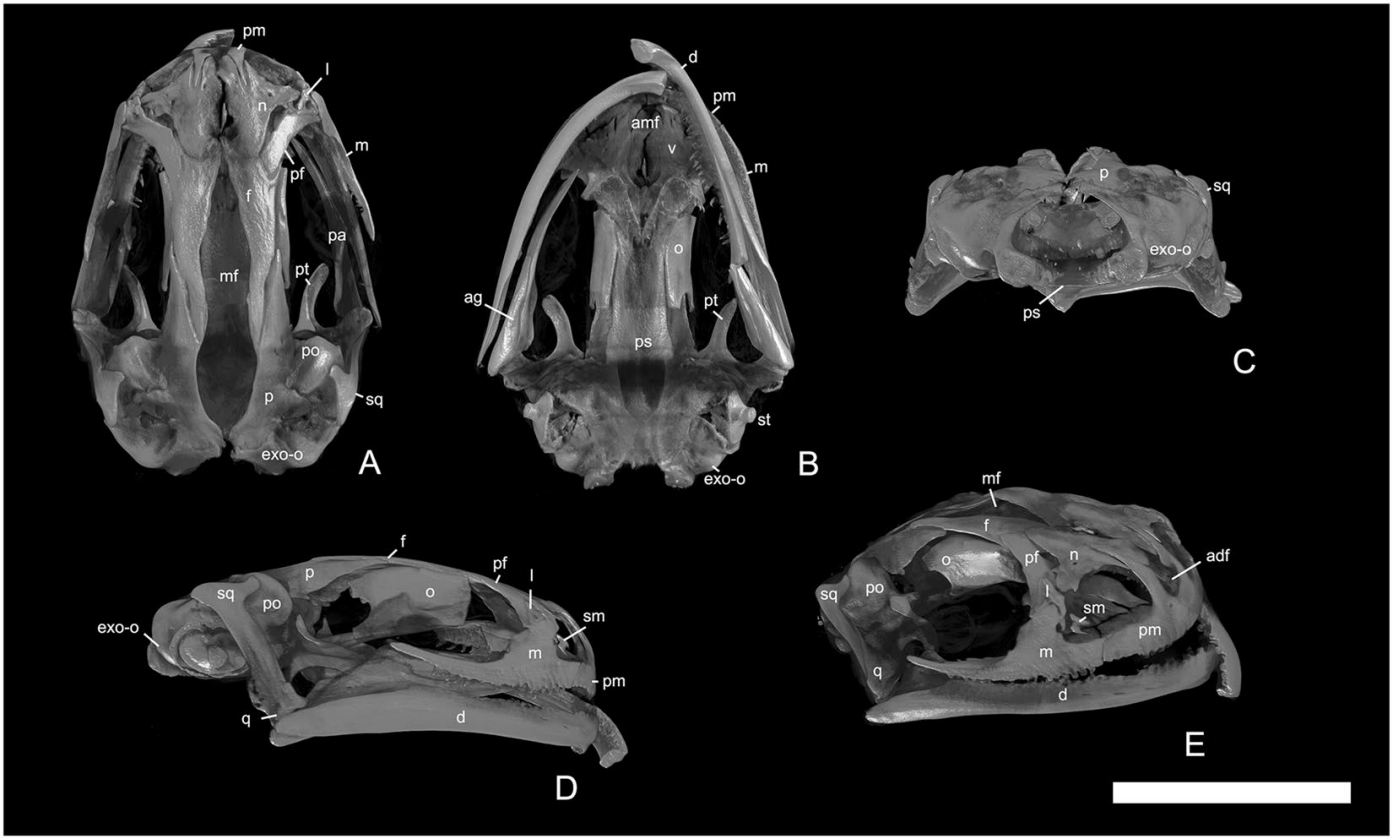
3D reconstruction of the skull of *Salamandrella keyserlingii* (specimen DVZ M 1/12). (**A**) In dorsal, (**B**) in ventral, (**C**) in posterior, (**D**) in lateral and (**E**) – in anterolateral views. Abbreviations: adf anterodorsal fenestra, ag angular, amf anteromedial fenestra, d dentary, exo-o opisthotic-exoccipital, f frontal, l lacrimal, m maxilla, mf median fontanella, n nasal, o orbitosphenoid, p parietal, pa prearticular, pf prefrontal, pm premaxilla, po prootic, ps parasphenoid, pt pterygoid, q quadrate, sm septomaxilla, sq squamosal, st stapes, v vomer. Scale bar 5 mm.

**Figure 2:**
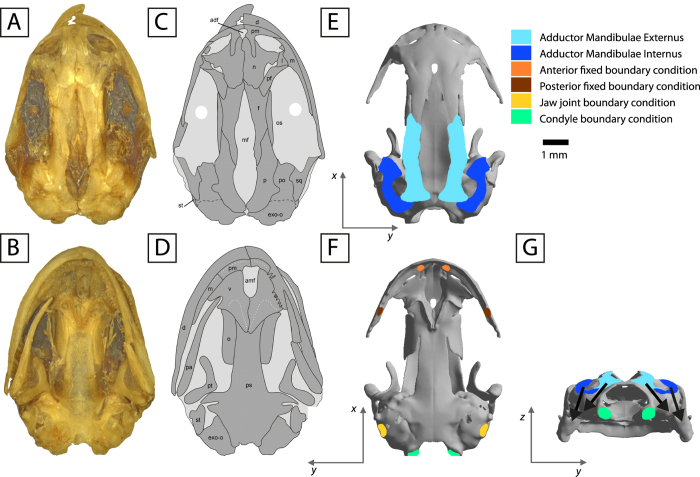
The skull of *Salamandrella keyserlingii* (specimen DVZ M 1/12). Photograph in dorsal view (**A**) and interpretative drawing (**C**). Photograph in ventral view (**B**) and interpretative drawing (**D)**. Loading and boundary conditions used to simulate bilateral prehension in dorsal (**E**), ventral (**F**) and posterior view (**G**). Dark grey areas in interpretative drawings represent bones and light grey areas represent connective tissues. Arrows in occipital view indicate direction of the force. Scale bar is 1 mm. Abbreviations as in Fig. 1.

The objectives pursued in this study are to (a) describe the feeding biomechanics of the adult Siberian salamander *Salamandrella keyserlingii*, a small, terrestrial Eurasian hynobiid (total length less than 12 cm, but see Kuzmin^25^ for size discussion); (b) discuss the biomechanical patterns that affects the skulls of the crown-group salamanders by comparing with the primitive cryptobranchids and derived groups of salamanders (e.g. dicamptodontids); and (c) discuss the implications of the ossification pattern of the skull roof for feeding biomechanics and its evolutionary and developmental implications. To achieve these goals, a postmetamorphic skull of *S*. *keyserlingii* was digitized and analyzed by 3D Finite Element Analysis (FEA) (Figs 1 and 2). FEA is a useful tool to test biomechanical hypotheses from an inductive or deductive perspective^26^; we used a hypothetical-deductive approach to simulate the differences found between bone and cartilage in the skull of *S. keyserlingii* and to qualitatively (distribution patterns) and quantitatively (stress values) investigate how the ossification sequence affect the internal forces (stress) experienced by the skull. Two loading cases, a bilateral prehension under two different prehension positions were analyzed: an anterior prehension (nearing the premaxilla-maxilla suture region) and a posterior prehension (on the most posterior part of the maxilla – most posterior teeth) (See Methods). To evaluate how ossification changes affect stress distribution and stress values, the area between the parietals and the frontals (the median fontanelle) (Figs 1 and 2) was simulated under different material properties or directly without material in this area (see Methods). It should be noted that broad cranial fontanelles in different regions of the skull, such as between parietals and frontals, even in large or old adults have been described as covered by connective tissue (collagen)^27^.

## Results

Distribution and values of Von Mises stresses were recorded for the different prehension positions (anterior and posterior) as well as under different mechanical properties in the skull roof. Differences in the distribution of stress patterns considering the two gape angles (21° and 6°) analyzed are negligible and described together, but see Supplementary Table S1 (for differences in stress values of the fontanelle), Supplementary Table S2 (for differences in stress values of the whole cranium), Supplementary Fig. S1 (for comparison of stress distribution). Estimated bite force on the prey when a prehension was made using the models was recorded, and the reaction appeared in the fixed boundary condition that simulates the prehension for the two gape angles was analyzed (Table 1).

**Table 1:** Analyzed cases in this study. Numerical results for cases 1*–*6 are presented in the Supplementary Tables S1 and S2. For cases 7*–*9 it can be found at the Supplementary Table S3 and for case 10 at the Supplementary Table S4.

Case	Taxon	Model source	Gape angle analyzed	Prehension position	Fontanelle	Varying material properties (Young’s modulus)	Ossification sequence
1	*Salamandrella keyserlingii*	Present study	6° and 21°	Anterior and posterior	No	6.65 GPa (Bone)	
2	*Salamandrella keyserlingii*	Present study	6° and 21°	Anterior and posterior	Yes	5.0 GPa	
3	*Salamandrella keyserlingii*	Present study	6° and 21°	Anterior and posterior	Yes	3.35 GPa	
4	*Salamandrella keyserlingii*	Present study	6° and 21°	Anterior and posterior	Yes	1.7 GPa	
5	*Salamandrella keyserlingii*	Present study	6° and 21°	Anterior and posterior	Yes	0.0665 GPa	
6	*Salamandrella keyserlingii*	Present study	6° and 21°	Anterior and posterior	Yes	No cranial material	
7	*Salamandrella keyserlingii*	Present study	6° and 21°	Anterior and posterior	Yes	6.0 GPa, 5.0 GPa, 3.35 GPa, 1.70 GPa, 0.065 GPa	Lateral to medial
8	*Salamandrella keyserlingii*	Present study	6° and 21°	Anterior and posterior	Yes	6.65 GPa, 5.0 GPa, 3.35 GPa, 1.70 GPa, 0.065 GPa	Caudal to Rostral
9	*Salamandrella keyserlingii*	Present study	6° and 21°	Anterior and posterior	Yes	6.65 GPa, 5.0 GPa, 3.35 GPa, 1.70 GPa, 0.065 GPa	Rostral to Caudal
10	*Dicamptodon ensatus*	Fortuny *et al*.^13^	21°	Anterior and posterior	No	No	No

### The anterior and posterior prehension

**Figure 3:**
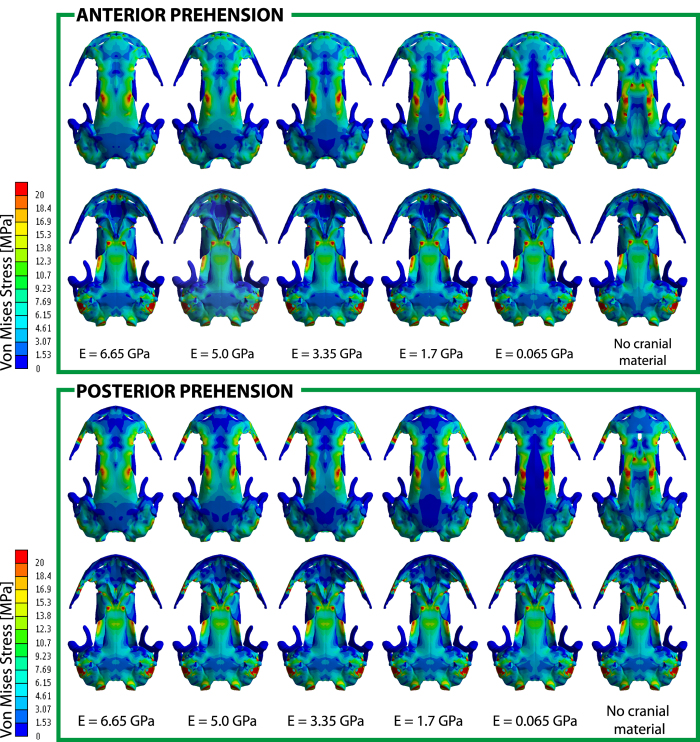
Von Mises stress results (in MPa) of bilateral loading cases under an anterior and posterior prehension, for a gape of 21°, and different material properties in the median fontanelle region (cases 1*–*6, from left to right). Top row in each panel is dorsal view and bottom row is ventral view.

The general stress pattern, under an anterior prehension (Fig. 3), in all the cases (different material properties in the analyzed region between parietals and frontals) were similar in the entire skull. Regarding the skull roof, the anterior part of the skull (premaxilla – maxilla – nasal) showed low (3*–*6 MPa) or very low (0*–*3 MPa) levels of stress, which increased around the prefrontal-nasal-frontal suture, but decreased between the frontals. However, the highest peak of stress in the whole cranium was found along the parietal-frontal suture in all the cases: higher stresses (18*–*20 Mpa) are present on the lateral margin borders of these two bones and conforms an area (resembling a belt) where high level of stress are present. This stress belt region continues across the palate (see below). In the posterior part of the skull roof, stress levels decreased, being very low in the posterior part of the parietals, and anterior part of the squamosals, and being moderate around the squamosal – exoccipital sutures.

Regarding the palate, the anterior part (premaxilla, maxilla and vomer) presented low (3*–*6 MPa) or very low (0*–*3 MPa) levels of stress, with areas of high stress in the premaxilla where the biting point was located, probably related to the boundary condition and consequently should not be considered (See Methods). Otherwise, high levels of stress were present on the most posterior part of the vomer (bordering with the parasphenoid and the orbitosphenoid). Also, as previously mentioned, a stress region resembling a belt, originated in the frontal-parietal suture, is found nearing the parasphenoid – orbitosphenoid, with moderate-high levels of stress. From the middle palate to the most posterior part of the palate, there was a general tendency to decrease the levels of stress (most of the parasphenoid). Very low (0*–*3 MPa) levels of stress were present in most of the squamosal, but increasing around the basicranial articulation, the central part of the squamosal and the exoccipital. In the latter bone, the levels of stress tended to increase, being moderated around the exoccipitals.

Regarding the analyses of a posterior prehension (Fig. 3), some differences in the general stress pattern were present: while the anterior part of the skull present low (3*–*6 MPa) or very low (0*–*3 MPa) levels of stress as in the anterior prehension, there was an important increase of stress in the maxilla (in comparison with an anterior prehension) originated by the boundary condition in this area under this loading case. Under this case the prefrontal also increases its level of stress in comparison with the anterior prehension one, but more importantly, it shows a general tendency of increasing stress levels from anterior (originated in the maxilla) to posterior (up to the parietal). Nonetheless, as in the anterior prehension, a stress belt was originated around the frontal-parietal suture and the stress levels decreased in the most posterior part of the skull except for an increasing around the squamosal – exoccipital suture. In the palate as a whole, the levels of stress were higher in comparison with the anterior prehension, particularly around the maxilla and the posterior part of the vomer. As in the anterior prehension, a stressed area resembling a belt, originated in the skull roof, and was present with moderate levels around the medium area of the parasphenoid and decreased in the posterior part of the parasphenoid. Similar to the anterior prehension, high levels of stress were present around the basicranial articulation, exoccipital and the central part of the squamosal.

### Effects of Bone and allied tissue in feeding biomechanics

**Figure 4:**
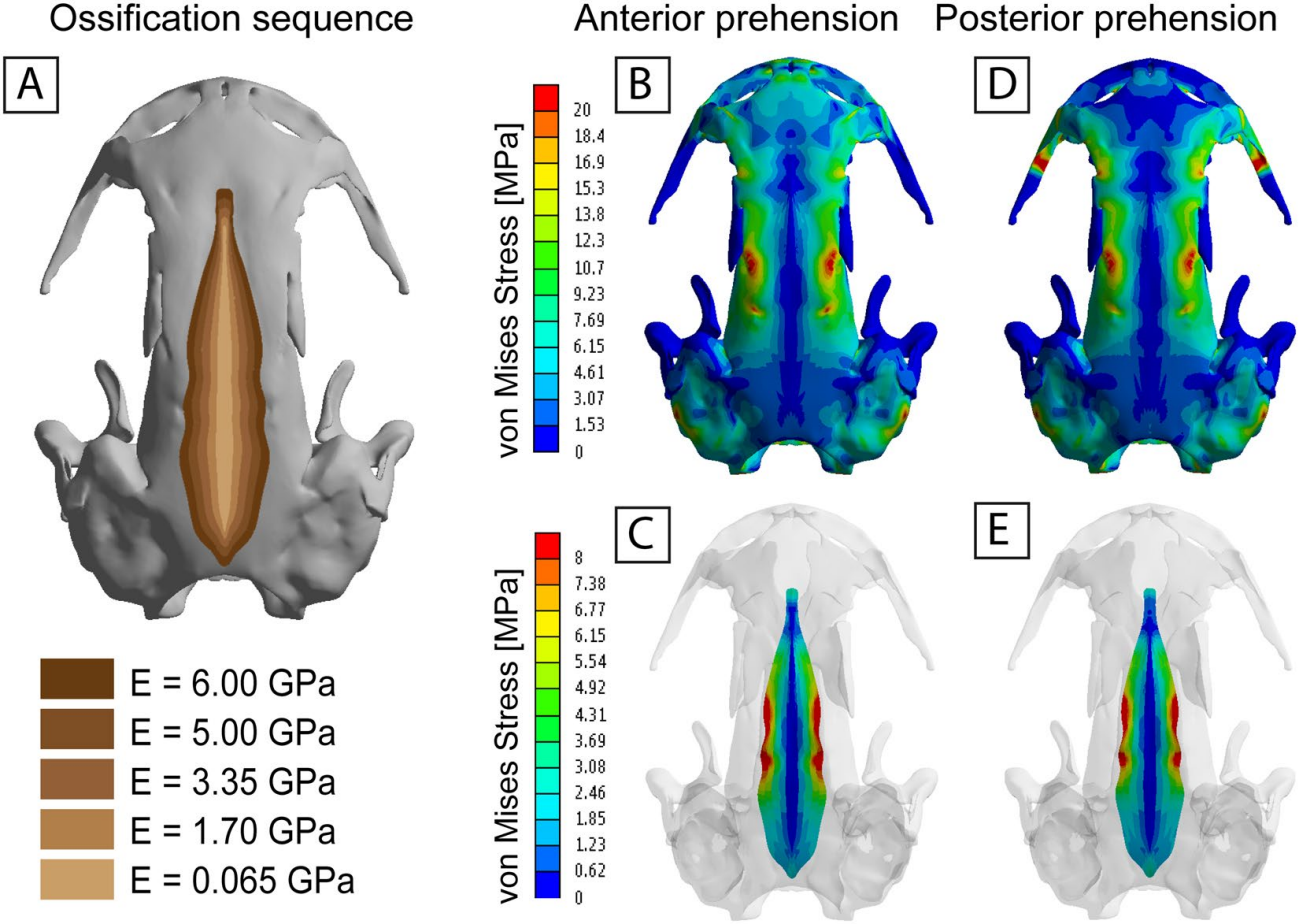
(**A**) Analysis of the ossification sequence in the median fontanelle: subdividing the median fontanelle in 5 sections from lateral to medial with different Young’s modulus values (decreasing Young’s modulus value from lateral to medial). Case 7 (See Supplementary Information for cases 8*–*9). Von Mises stress results (in MPa) under an anterior (**B**,**C**) and posterior prehension (**D**,**E**). Figures C and E only show the median fontanelle region for proper visualization.

**Figure 5:**
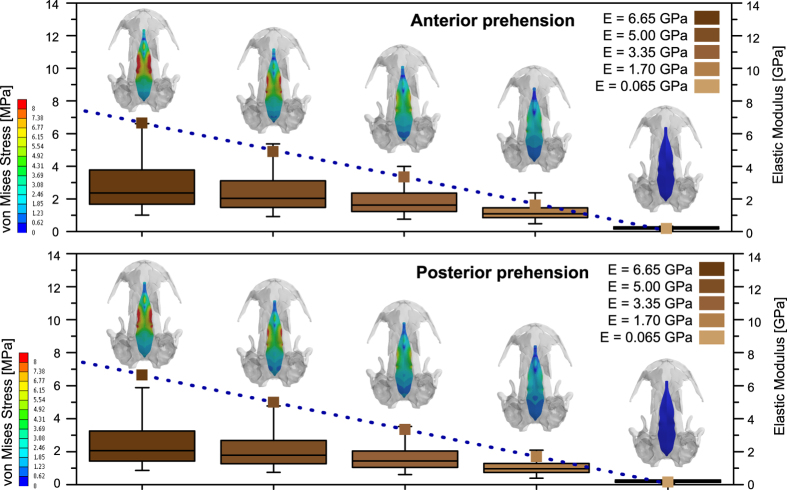
Box-plot results (cases 1*–*6) under an anterior and posterior prehension using 5 different Young’s modulus values in the whole median fontanelle region (not subdividing it) to evaluate the ossification pattern of this region.

The Von Mises Stress obtained when the value of the Young’s modulus is changed (Figs 3, 4 and 5, Supplementary Figs S1, S2, S3, Table 1, Supplementary Table S1) shows that changes in the mechanical properties of regions that present cartilage, herein the median fontanelle, don’t affect the strength of the skull bones, but produce differences in the stress state and elasticity of this median fontanelle. In other words, the more elastic the median fontanelle is (low Young’s modulus), the lower values of Von Mises stress are found in this cartilaginous region, and the fracture of the material is more difficult to reach. On the other hand, the stiffer the median fontanelle (high Young’s modulus), higher values of stress and will appear, leading to an easier fracture of the material.

However, this elasticity of the fontanelle implies more flexibility and mobility of this region (further deformation), and in consequence, the displacements between parietals and frontals (which surround the median fontanelle) are easier in comparison with other areas more constrained. The impact of these changes in the skeletal material implies a consensus between the stiffness required for the function of the bone and the required fixation with the rest of the bones. For the same level of deformation, the stresses in more elastic materials will be lower.

The results in both anterior and posterior prehensions show a similar trend to decrease the levels of stress when the Young’s modulus of the cartilage decreases (Figs 3, 4 and 5, Supplementary Figs S1, S2, S3). On the one hand, when tested the ossification sequence in the fontanelle from lateral to medial (Fig. 4, Supplementary Table S3), the stress distribution reveals higher stress levels laterally but reducing medially, with no stress in the medial region. This stress tendency decreases when the stiffness of the cartilage is reduced, being low to no stress in the analyzed area when the stiffness is reduced almost four times and the stress in the cartilage is completely absent under lower stiffness.

When the ossification pattern is considered using different material properties in the median fontanelle as a whole, from higher to lower Young’s modulus (a decreasing stiffness) (Fig. 5), the results show that both the anterior and posterior extremities of the fontanelle present low or very low levels of stress. But in the central part (coinciding with the area of peak levels of stress) the stress is present but reduced in comparison with the cases where the stiffness is homogeneous in the whole median fontanelle. Alternative ossification sequences from caudal to rostral (or reverse direction) also shows similar patterns (Supplementary Figs S2, S3, Table S3).

## Discussion

The Siberian salamander, as well as other modern hynobiids^6, 28^, retain many cranial morphological features that are widely distributed among more basal Middle Jurassic-Early Cretaceous crown-group taxa. These features, presumably plesiomorphic, include: similar composition of skull roof bones (including the presence of lacrimal bone); similar composition of palate and braincase bones; presence of the anteromedial fenestra in the palate and the anterodorsal (=internarial) fenestra between premaxillae (see descriptions and figures of the skulls of the Middle Jurassic-Early Cretaceous crown-group taxa^24, 29–37^). These similarities make modern hynobiids a potentially good model for the reconstruction of feeding biomechanics in most of Mesozoic basal crown-group salamanders, except taxa with very different proportions for their skulls (the Chinese Middle Jurassic cryptobranchoid *Pangerpeton* ^38^), highly paedomorphic skulls (the Chinese Middle Jurassic possible cryptobranchoid *Jeholotriton* ^39, 40^) and taxa with principally different patterns of fenestration (the European Early Cretaceous salamandroid *Valdotriton* ^41^).

The main difference between the skull of *S. keyserlingii* and that of most Mesozoic crown-group salamanders is the presence of the fontanelle between parietals and frontals (=median fontanelle). The fontanelle between parietals and frontals was described only for one Middle Jurassic crown salamander – the possible cryptobranchoid *Jeholotriton paradoxus* from China^39, 40^. *Jeholotriton* was a neotenic and aquatic salamander with many larval features retained in the skull (versus fully metamorphosed skull in the Siberian salamander). The median fontanelle between parietals and frontals in *Jeholotriton* is much smaller than that of *S. keyserlingii*. In another fossil taxon – *Beiyanerpeton jianpingensis* (the oldest salamandroid, Late Jurassic, China), it is possible to see a small and narrow median fontanelle on the interpretative drawing of the skull (see Gao and Shubin^32^: Fig. 3), but the presence of the median fontanelle in this taxon needs a further confirmation.

The skulls of *S. keyserlingii* and other modern hynobiids differ from that of cryptobranchids in many aspects. Skulls of modern cryptobranchids are larger, more robust and wider than that of hynobiids. In cryptobranchids, the anteromedial fenestra in the palate and the anterodorsal fenestra between premaxillae are absent, the frontals contact with the maxillae and the parietals are strongly overlapped by the frontals^18, 31^.Also, the biomechanical patterns of the skulls of the Siberian salamander and modern cryptobranchids (*Andrias*) are different: Cryptobranchids, and *Andrias* in particular, are well known for its capability to perform asymmetrical feeding (autopomorphic feature of this group)^12, 18, 42^. In *Andrias*, significant differences of stress distribution between anterior and posterior prehension are present^12^, whereas in *S. keyserlingii* the differences between the position of the prehension are present, but much less marked. In both cases, a stress belt is present but is located in an anterior position in the *Andrias* case (around the nasal-frontal suture)^12^, and posteriorly moved in *Salamandrella* (around parietal-frontal suture). Comparison of the biomechanical patterns of skulls of the Siberian salamander and derived modern crown-group salamander *Dicamptodon ensatus* (Dicamptodontidae) revealed some similarities described by the following, as well as in comparison with the genus *Andrias*: under an anterior and posterior prehension, differences between the two cases are present but not so marked (contrary to marked differences found in *Andrias*)^12, 13^ (Supplementary Fig. S4). The stress belt found in *Andrias* and *S. keyserlingii* is much less marked in *Dicamptodon*, but present in this latter genus. Morever, as found in *Salamandrella* this stress belt is around the parietal-frontal suture in *Dicamptodon* ^13^ (Supplementary Fig. S4). However, in all three taxa, high stress levels are found in the posterior part of the skull around the squamosal-quadrate-exoccipital bones in all the cases^12, 13^ (Supplementary Fig. S4).

The biomechanical pattern found in dicamptodontids and its similarities with hynobiids suggest that other factors (i.e. lifestyle, convergence) partly mask the phylogenetic signal, but are key to the adaptation of the different crown-group salamanders to their environments.

Among modern salamanders, the median fontanelle is present in some hynobiids (e.g., *Ranodon* ^28^) but also in several plethodontids (e.g. *Oedipina uniformis*, *Batrachoseps* and *Thorius* ^27, 43^. The presence/absence of the median fontanelle in hynobiids does not correlate with a lifestyle, because it could be present in both terrestrial (*Salamandrella keyserlingii*) and in aquatic (but metamorphosed) forms (*Ranodon*). Our results show that the presence of the median fontanelle filled by connective tissue does not affect the strength of the whole skull bone, but changes in the stiffness produce further mobility (and deformation) on the surrounding bones. The appearance of the median fontanelle in some hynobiids is not caused by rearrangement of adductor muscles (the pattern of adductor muscles attachment in *Salamandrella* is the same with other hynobiids without the median fontanelle)^4, 6, 44^, so, the potential functions and implications for the presence of fontanelle should be considered from a developmental (among others, out of the scope of the present study) points of view.

From a developmental point of view, the appearance of fontanelle may be related with bone loss without implying significant differences on the strength of the skull during biomechanical loadings (Supplementary Table S2). This bone loss might be a consequence of a biomechanical optimization possibly implying a reduction of developmental costs, with potential benefits for an ectothermic animal living on extreme conditions (the most ectothermic terrestrial vertebrate in Eurasia in the case of *Salamandrella*). Bone loss in early urodeles has been discussed for the cranial simplification present in batrachians in comparison with extinct groups^3^. This cranial simplification trend is also apparent in the lack of a bony bridge (as quadratojugal and jugal) between maxilla and squamosal/quadrate, found in extinct early amphibians groups as dissorophoids (particularly branchiosaurids). This bony bridge is rare in living salamander groups, present in *Echinotriton* and *Tylototriton* but also in some salamandrids. Another structure, cartilage bridge (jugal ligament)^18, 45^ between maxilla and pterygoid, is present in some hynobiids (e.g. *Batrachuperus pinchonii*)^21^ and potentially could act as a stabilizer of the skull. The bony bridge (jugal/quadratojugal bone) and cartilage bridge (jugal ligament) are not homologous, but the bony bridge function to stabilize the skull when present and its loss is potentially derived from the cranial simplification. Nonetheless, in taxa without this bony bridge (jugal/quadratojugal), the cartilage bridge (jugal ligament) plays the same role as the bony bridge to stabilize the skull. Otherwise, as previously mentioned for *Salamandrella*’s fontanelle, these changes on the ossification pattern (or directly failure on ossification) imply a major mobility on the suture region, potentially helping to distribute stress through the skull but, at the same time, could imply a major destabilization of the skull. Taking into account these considerations, *Salamandrella* (and hynobiids in general) resemble extinct early amphibian groups (e.g. dissorophoids) and early urodeles but with a simplified cranial structure that, at least for some structures as fontanelles, revealed few changes on the strength of the skull.

This study adds to a growing number of studies that focus on computational biomechanics to test inductive-deductive hypotheses that develop the relationship between form and function. This kind of study shows how computational biomechanics can shed light on evolutionary history as well as on development and ossification sequence studies. In batrachians, particularly in salamanders, computational biomechanics sheds light not only on biomechanical patterns on crown-group salamanders but also deciphers evolutionary and developmental patterns in early history of urodeles and related early amphibian groups.

## Methods

### Sample and digitization

*Salamandrella keyserlingii* is the most widespread ectothermic terrestrial vertebrate in Eurasia, occupying the widest range compared to other Paleartic salamanders, extending from the Hokkaido Islands (Japan) and Sakhalin (Russia); Kamchatka to eastern European Russia, south to northern Mongolia, northeastern China and Korea^21, 46–48^. The number of species included in the genus *Salamandrella* is controversial, as some authors consider only one species valid, *S. keyserlingii*, (considering extreme morphological uniformity without a distinct geographical variation^49^) but others consider the validity of a second species *S. tridactyla* (See Frost^21^ for a discussion).

In the present study, a specimen of *S. keyserlingii* with a skull length of 9 mm, stored in the morphological collection of the Department of Vertebrate Zoology of the Saint Petersburg State University, Saint Petersburg, Russia (specimen DVZ M 1/12), was used to perform the 3D analyses (Figs 1 and 2). The precise age and locality of the specimen are unknown. The relatively large size of the skull (9 mm) and the presence of bones which ossify during or after metamorphosis in hynobiids (e.g., septomaxilla) demonstrate that the individual represents an adult.

The skull was CT scanned at the “Geomodel” Research Center at the Saint Petersburg State University (Saint Petersburg, Russia) using CT model Skyscan 1172. *Salamandrella keyserlingii* was scanned at 47 kV and 159 mA, generating a resolution of 4.62 µm of pixel size and an output of 1536 × 1009 pixels per slice. CT scan data was imported to the software Avizo 7.0 (FEI-VSG Company), where the model was reconstructed and segmented (Fig. 2). The segmented model was then converted into a CAD model^50^. During this last step, irregularities in the surface caused by segmentation were repaired using refinement and smoothing tools.

### Model Properties

A Structural Static Analysis to evaluate the biomechanical behavior of the skull and the role of the adductor musculature (see below) was performed using the Finite Element Package ANSYS 17.1 in a Dell Precision™ Workstation T5500 with 48 GB and 5.33 GHz.

The cranial bone mechanical properties for living salamanders or any other anamniote are unknown, but well known for many groups of mammals and reptiles. The mechanical properties of bone allied tissues, such as cartilage, connective tissue or collagen, are difficult to decide because they must take into account viscoelastic properties^51^. An attempt to measure the elastic modulus of calcified cartilage in humans (collagen mineralized with hydroxyapatite) revealed calcified cartilage was considerably less stiff than the subchondral bone (0.35 and 5.7 GPa, respectively)^52^.

In the present study elastic, linear and homogeneous material properties were assumed for the bone, using the following mechanical properties from the frontal and prefrontal of *Crocodylus*: E (Young’s modulus) 6.65 GPa and v (Poisson’s ratio) 0.35^53^, while the area between parietals and frontals (median fontanelle) was analyzed under 5 different Young’s moduli, defined between the bone value (6.65 GPa) and a low value close to zero. The values were equally distributed. Unfortunately, real Young’s modulus values for amphibians are not available in the literature. The aim was to change the stiffness of the cartilage to test the impact on skull mechanics. From low stiffness to high stiffness: 0.0665 GPa, 1.7 GPa, 3.35 GPa, 5.0 GPa and 6.65 GPa (same value as the rest of the bone model) (Table 1). This range of Young’s moduli values allows us to evaluate how stress patterns affect the stiffness, but not the strength, of the material and values change during the ossification process and how skull mechanics are impacted during the developmental sequence. Moreover, as the skull roof bones, in *S. keyserlingii* and the closely related hynobiid *Ranodon* develop and ossify sequentially from anterior to posterior. Frontals and parietals in these two taxa approach each other medially^4, 28^. Thus, the median fontanelle of *S. keyserlingii* is supposed to be closed following a lateral-to-medial direction. For this reason we evaluated the affect to the skull mechanics if the analyzed area between parietals and frontals is subdivided in 5 sections from lateral to medial giving to each section different modulus values (decreasing Young’s moduli value from lateral to medial): 0.0665 GPa, 1.7 GPa, 3.35 GPa, 5.0 GPa and 6.0 GPa (Fig. 4, Table 1, Supplementary Table S3). We additionally also tested two alternative scenarios subdividing the median fontanelle in 5 sections, but tested how results changed if skull bones ossified from rostral to caudal (Supplementary Fig. S2, Table S3) or caudal to rostral (Supplementary Fig. S3, Table S3). This latter case is found in plethodontids^27^ clearly differing in many aspects from hynobiids.

Potential orthotropic properties were not modelled. Orthotropic and heterogeneous material properties have an impact to the results of skull mechanics. Nonetheless, in a comparative analysis, as the present one, the use of the same linear and homogeneous material properties for different specimens has been demonstrated to be useful for studying biological questions^26, 54–56^ and, according to the sensitivity analysis of Walmsley *et al*.^55^, results for heterogeneous properties of the bone closely match the results for homogeneous properties (See Strait *et al*.^56^ for further discussion on material properties in biological models). The cranium model (but not the jaw) was meshed with a regular mesh of 10-noded tetrahedral elements^50^ to facilitate the statistical post-process of the results (see below). The mesh of the analyzed specimen was approximately 1.6 million elements and 2.4 million nodes

### Boundary and loading conditions

Adductor musculature was added to the model: adductor mandibulae externus (AME) and adductor mandibulae internus (AMI). The adductor mandibulae posterior (AMP) is small and usually poorly differentiated in salamanders^44^ and was not considered in this study. The origin and insertion of each muscle have been largely described^1, 14, 17, 44^. Areas of muscular insertion of AME and AMI were considered following previous anatomical studies in hynobiids and particularly in *Salamandrella* ^3, 17, 57^ and references therein). These areas of muscular insertion were defined in the cranial geometric model to apply the forces of the muscular contraction during the prehension (Fig. 2). The direction of these forces was defined by the line that joins the centroid of the insertion area in the cranium with its insertion on the jaw. A gape of 21° was assumed. This gape represents the maximum gape angle, considering 6° of elevation of the skull and a depression of the mandible of 15° as reported in other basal salamanders^14^. Previous studies demonstrated the low influence in stress distribution using different gape angles^12, 13^. Nonetheless, a gape angle of 6° was also tested (See Supplementary Fig. S1, Table 1). A value of 0.3 MPa (force per unit area) was assumed as muscular contraction pressure in AMI and AME following^58^. The muscle attachment in the cranium was used as a Cross-Sectional Area (CSA). This assumption allows the application of the muscle pressure directly to the area of its muscular insertion.

Two loading cases were analyzed: a bilateral prehension (=bite) simulating that the two sides of the skull were biting at same time under two different prehension positions (anterior and posterior). The bilateral prehension was simulated in two different positions: an anterior position, near the premaxilla-maxilla suture region, and a posterior prehension position on the most posterior part of the maxilla (most posterior teeth) (Fig. 2). Asymmetrical biting (unilateral prehension in which only one side of the skull is biting and the other side is the balancing side) was not considered as hynobiids (and most salamanders with the exception of giant salamanders) don’t perform this type of biting^12, 18, 42^. For these purposes, a fixed boundary condition on a surface was applied in the three dimensions in these two locations (anterior and posterior) to simulate the moment that skull and mandibles contact the prey (Fig. 2). Moreover, in all the cases constraints (a surface for each constraint) were applied at the jaw joint (the quadrate) in z-direction (simulating contact of the cranium with the jaw) and at the double-headed occipital condyle in the x-direction (simulating the attachment of the vertebral column) (Fig. 2).

It should be remarked that unusual high stress values (peak values) appear where the boundary conditions are set as a simple support or where we have an abrupt change in the material properties, geometry, etc. These stresses are artificially inflated by the constraints imposed on the model due to a numerical singularity. The numerical singularity is a consequence of the mathematical approach, and it is not related to any biological process. In those areas stresses have the tendency to increase the value towards infinity when we are reducing the size of the elements of the mesh; therefore results of these areas should not be considered. For this reason in the present study we use the 95% value of the boxplot instead the peak value to study the maximums values of stress (See Marcé-Nogué *et al*.^59^ for further details).

For additional comparisons, a FE model of *Dicamptodon ensatus*, recently published by Fortuny *et al*.^13^ was scaled to compare the performances of skulls that differ in shape and size.

For this reason, we scaled the values of muscular contraction pressure of *D. ensatus* in order to be able to compare Von Mises stress following Dumont *et al*.^60^ and scaled by surface area.

Two different cases of *Dicamptodon ensatus* were calculated using in each one a different reference *Salamandrella* model: one considering the presence of a median fontanelle and another without it (Supplementary Fig. S4 and Supplementary Table S4).

### Statistical Analysis

The Von Mises stress distribution of the median fontanelle was analyzed using the average values of Von Mises Stress and presented using box-plots of the stress distribution. According to Marcé-Nogué *et al*.^59^, and following the idea of Dumont *et al*.^61^, the use of box-plots for the stress and statistics derived of them (such as percentiles or whiskers) implies the use of a Quasi-Ideal Mesh (QIM) or implies corrections due to the non-uniformity of the mesh when accounting average values such as the Mesh-Weighted Average Mean (MWAM) and the Mesh-Weighted Mean (MWM) (Supplementary Tables S1, S2) Herein, box-plots were used to analyze how affect to the stress values changes in the Young’s modulus of the whole median fontanelle (Fig. 5).

## Electronic supplementary material

Supplementary information
