# Supplementary material for: Cranial biomechanics in basal urodeles: the Siberian salamander (Salamandrella keyserlingii) and its evolutionary and developmental implications
Source: Sci Rep. 2017 Aug 31;7:10174. doi: 10.1038/s41598-017-10553-1 (PMC5579059; doi:10.1038/s41598-017-10553-1)
Supplement: Supplementary file 1 — Supplementary information [file 41598_2017_10553_MOESM1_ESM.pdf]

Cranial biomechanics in basal urodeles: the Siberian salamander (*Salamandrella keyserlingii*) and its evolutionary and developmental implications

Zupeng Zhou, Josep Fortuny, Jordi Marcé-Nogué, Pavel P. Skutschas

*corresponding author: josep.fortuny@icp.cat

Supplementary Information

Supplementary Figures S1, S2, S3, S4

Supplementary Tables S1, S2, S3, S4

Supplementary Figure S1. Von Mises stress results (in MPa) of *Salamandrella keyserlingii* under bilateral loading and two different gape angles (6º and 21º) during an anterior and posterior prehension.

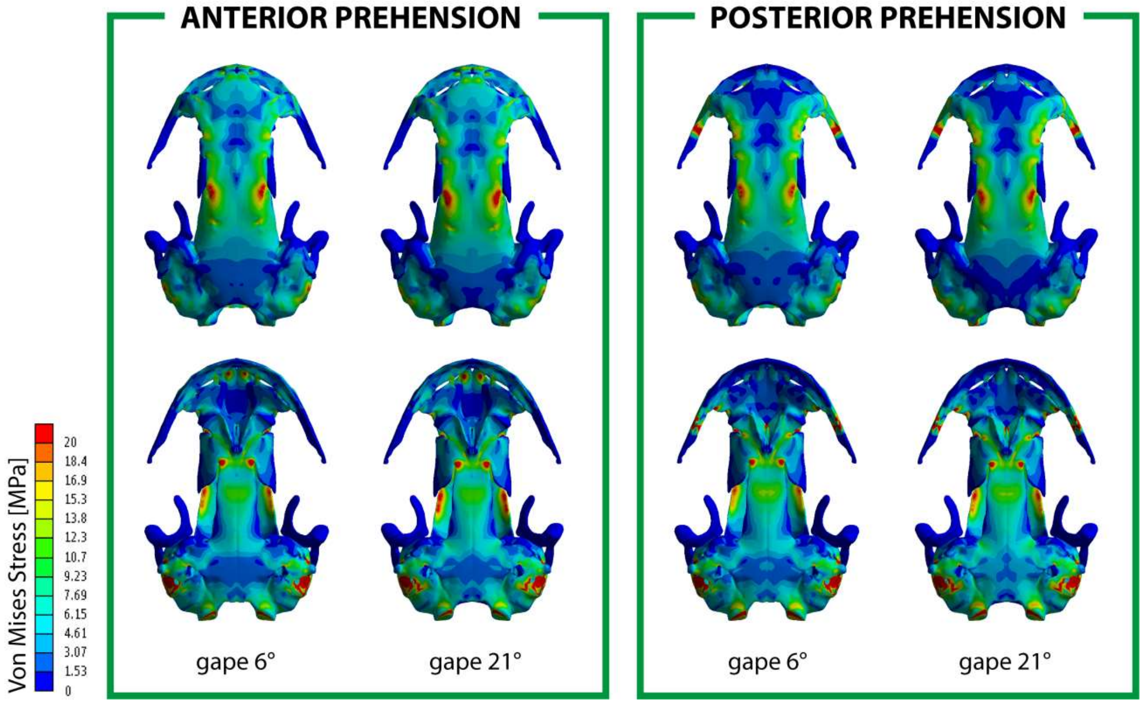

Supplementary Figure S2. (A) Analysis of the ossification sequence in the median fontanelle: subdividing the median fontanelle in 5 sections from caudal to rostral with different Young's modulus values (decreasing Young's modulus value from caudal to rostral). Case 8. Von Mises stress results (in MPa) under an anterior (B, C) and posterior prehension (D, E). Figures C and E only show the median fontanelle region for proper visualization.

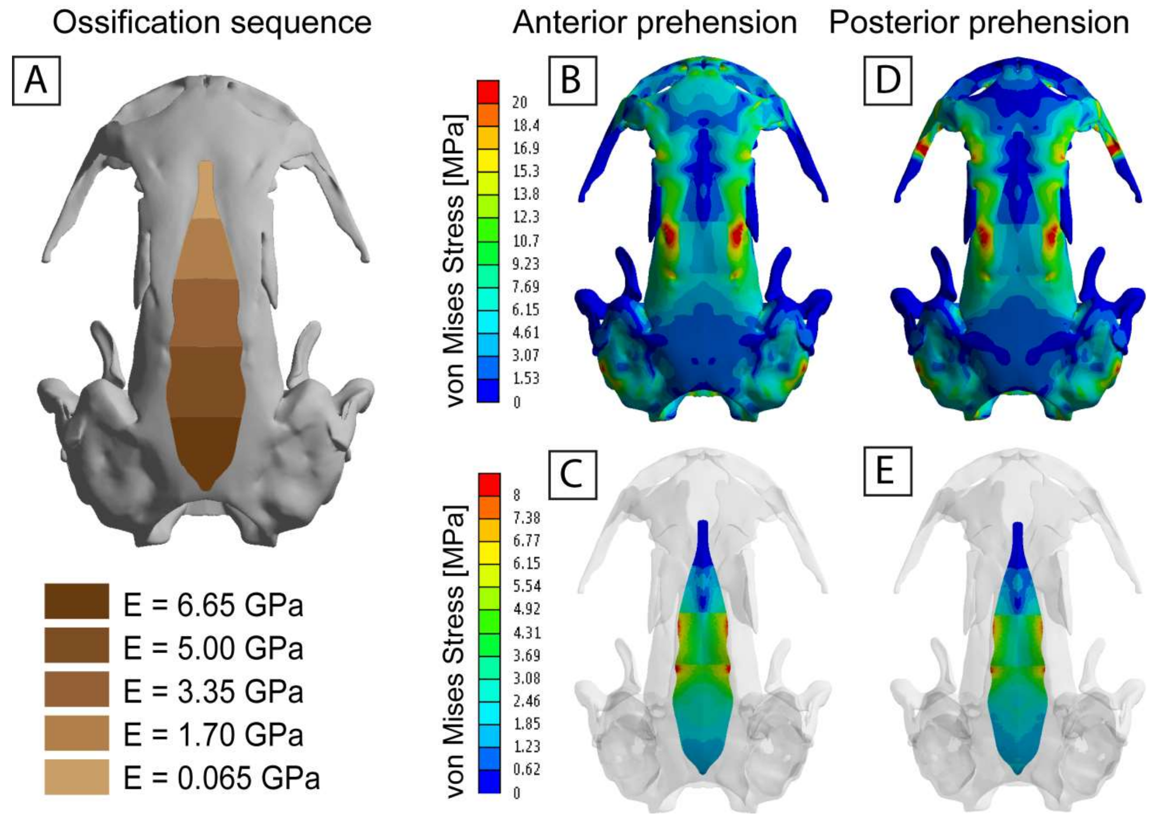

Supplementary Figure S3. (A) Analysis of the ossification sequence in the median fontanelle: subdividing the median fontanelle in 5 sections from rostral to caudal with different Young's modulus values (decreasing Young's modulus value from rostral to caudal). Case 9. Von Mises stress results (in MPa) under an anterior (B, C) and posterior prehension (D, E). Figures C and E only show the median fontanelle region for proper visualization.

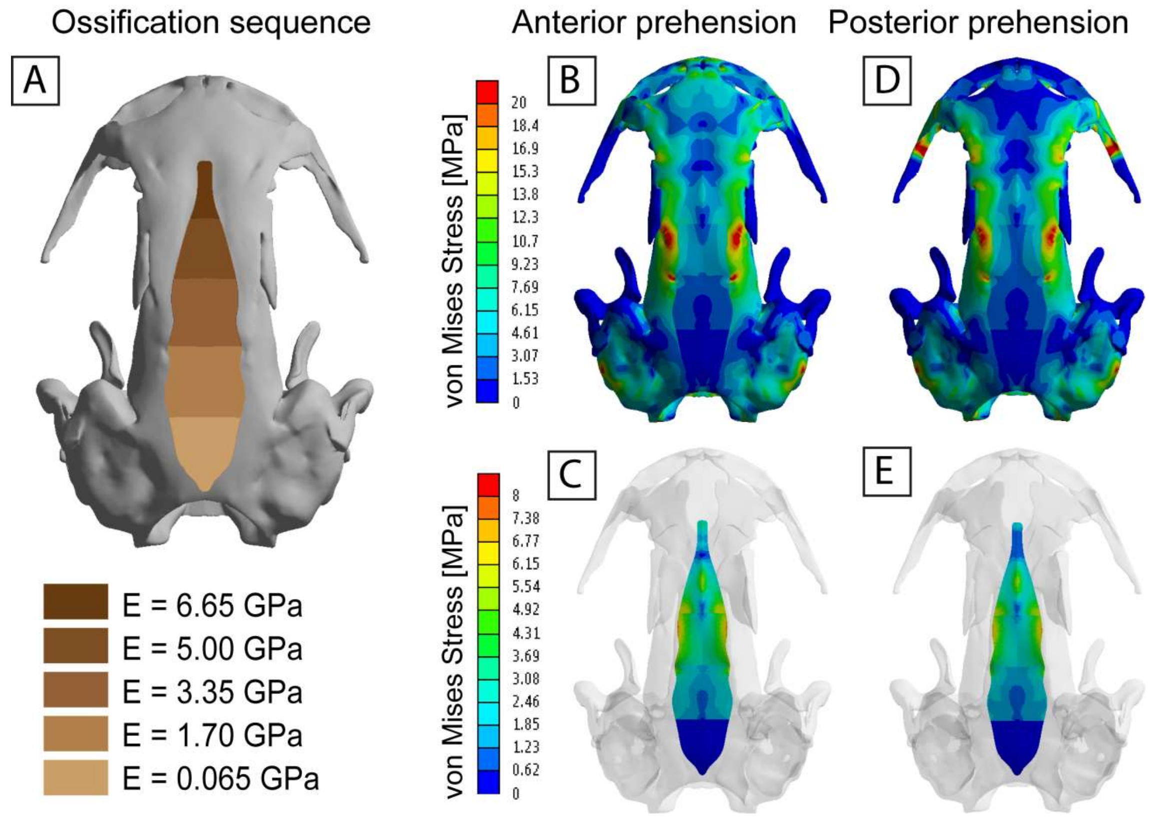

Supplementary Figure S4. Von Mises stress results (in MPa) of *Dicamptodon ensatus*. Bilateral loading case scaled using *S. keyserlingii* as reference model and a surface area approach. Case 10. Anterior and posterior prehensions tested. Two cases different cases of *D. ensatus* were calculated using in each one a different reference *S. keyserlingii* model: one considering the presence of a median fontanelle and another without the fontanelle (Supplementary Table 1).

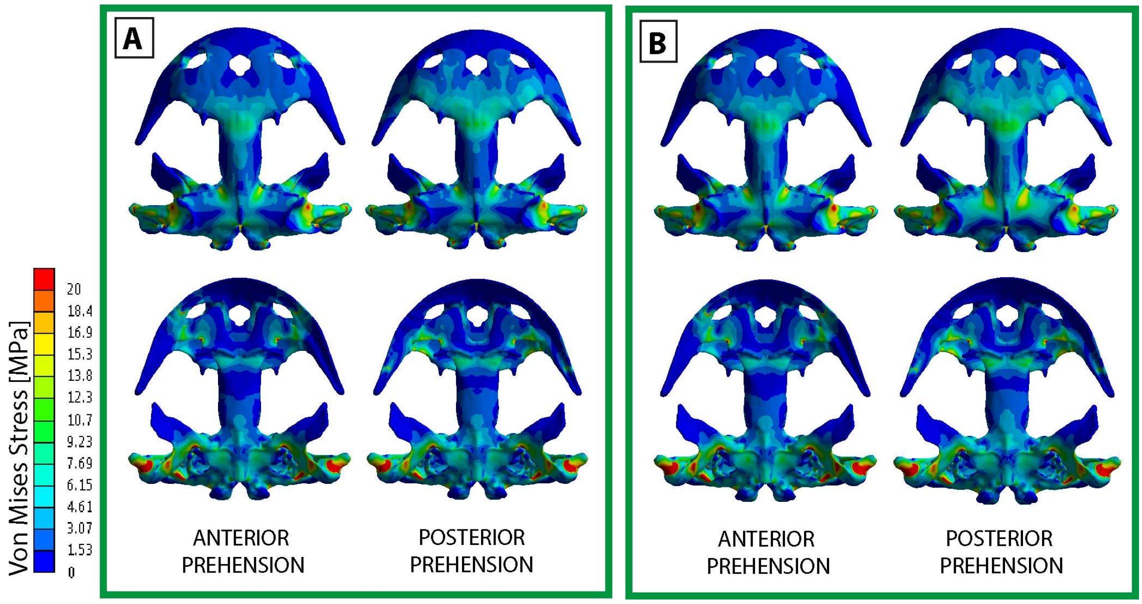

Supplementary Table S1. Results for anterior and posterior prehension with gapes of 21° and 6° during the parameterization of the Young's modulus (E = 0.0665 GPa, 1.7 GPa, 3.35GPa, 5.0 GPa and 6.65 GPa), in correlation with figure 5 (only fontanelle considered), and including the case without fontanelle (no cranial material) and the case where the ossification sequence was analyzed. MWAM: Mesh-Weighted Arithmetic Mean; MWM: Mesh-Weighted Median; PEofAM: Percentage Error of the Arithmetic Mean; PEofM: Percentage of Error of the Median. M: Median with subsequent quartile values. MWAM, MWM, PEofAM, PEofM are quality indicators of the mesh. M are numerical values in the quartiles of the boxplots. Bite force in Newtons (N).

GAPE 21°											
**ANTERIOR PREHENSION**	**E [GPa]**	**Elements**	**MWAM**	**MWM**	**PEofAM**	**PEofM**	**M(25%)**	**M(50%)**	**M(75%)**	**M(95%)**	**Bite Force [N]**
Case 1	6.65	96063	2.9248	2.3413	0.4903	1.1175	1.6699	2.3675	3.7676	6.6168	0.4872
Case 2	5	96063	2.4598	2.0127	0.4187	1.0381	1.4807	2.0336	3.1105	5.3740	0.4826
Case 3	3.35	96063	1.9080	1.6066	0.3329	1.3215	1.2264	1.6278	2.3546	3.9863	0.4767
Case 4	1.7	96063	1.2101	1.0644	0.2275	2.0307	0.8549	1.0860	1.4538	2.3686	0.4689
Case 5	0.065	96063	0.1985	0.1908	0.1499	2.6993	0.1582	0.1959	0.2367	0.3286	0.4568
Case 6 - No cranial material											0.4515
**POSTERIOR PREHENSION**	**E [GPa]**	**Elements**	**MWAM**	**MWM**	**PEofAM**	**PEofM**	**M(25%)**	**M(50%)**	**M(75%)**	**M(95%)**	**Bite Force [N]**
Case 1	6.65	96063	2.5510	2.0314	0.6265	1.4051	1.4294	2.0599	3.2468	5.8790	0.3389
Case 2	5	96063	2.1466	1.7557	0.5372	1.6724	1.2664	1.7851	2.6844	4.7733	0.3364
Case 3	3.35	96063	1.6691	1.4112	0.4270	1.9969	1.0513	1.4394	2.0429	3.5303	0.3333
Case 4	1.7	96063	1.0671	0.9475	0.2861	2.0491	0.7409	0.9669	1.2844	2.0888	0.3292
Case 5	0.065	96063	0.1792	0.1835	0.1043	3.7426	0.1415	0.1904	0.2186	0.2735	0.3217
Case 6 - No cranial material											0.3187

GAPE 6°											
**ANTERIOR PREHENSION**	**E [GPa]**	**Elements**	**MWAM**	**MWM**	**PEofAM**	**PEofM**	**M(25%)**	**M(50%)**	**M(75%)**	**M(95%)**	**Bite Force [N]**
Case 1	6.65	96752	3.1597	2.5262	0.4055	2.3928	1.7968	2.5866	4.1071	6.9926	0.5167
Case 2	5	96752	2.6635	2.1731	0.3868	2.7542	1.5930	2.2330	3.4151	5.6835	0.5107
Case 3	3.35	96752	2.0738	1.7421	0.3657	3.0452	1.3248	1.7952	2.6127	4.2186	0.5033
Case 4	1.7	96752	1.3252	1.1689	0.3415	3.5934	0.9203	1.2109	1.6373	2.4946	0.4935
Case 5	0.065	96752	0.2374	0.2268	0.2418	4.8966	0.1842	0.2379	0.2898	0.3813	0.4762
Case 6 - No cranial material											0.4656
**POSTERIOR PREHENSION**	**E [GPa]**	**Elements**	**MWAM**	**MWM**	**PEofAM**	**PEofM**	**M(25%)**	**M(50%)**	**M(75%)**	**M(95%)**	**Bite Force [N]**
Case 1	6.65	96752	2.6471	2.1109	0.4598	3.3947	1.4974	2.1826	3.3889	5.9944	0.3469
Case 2	5	96752	2.2319	1.8211	0.4336	3.8169	1.3294	1.8906	2.8181	4.8689	0.3442
Case 3	3.35	96752	1.7413	1.4664	0.4033	3.9655	1.1067	1.5245	2.1687	3.5989	0.3407
Case 4	1.7	96752	1.1211	0.9919	0.3631	4.3002	0.7635	1.0346	1.3964	2.1264	0.3361
Case 5	0.065	96752	0.2057	0.2048	0.2052	6.2950	0.1587	0.2177	0.2558	0.3174	0.3277
Case 6 - No cranial material											0.3237

Supplementary Table S2. Results for anterior and posterior prehension with gapes of 21° and 6° during the parameterization of the Young's modulus (E = 0.0665 GPa, 1.7 GPa, 3.35GPa, 5.0 GPa and 6.65 GPa) of the whole cranium and including the case without fontanelle (no cranial material). MWAM: Mesh-Weighted Arithmetic Mean; MWM: Mesh-Weighted Median; PEofAM: Percentage Error of the Arithmetic Mean; PEofM: Percentage of Error of the Median. M: Median with subsequent quartile values. MWAM, MWM, PEofAM, PEofM are quality indicators of the mesh. M are numerical values in the quartiles of the boxplots. Bite force in Newtons (N).

GAPE 21°											
**ANTERIOR PREHENSION**	**E [GPa]**	**Elements**	**MWAM**	**MWM**	**PEofAM**	**PEofM**	**M(25%)**	**M(50%)**	**M(75%)**	**M(95%)**	**Bite Force [N]**
Case 1	6.65	1495627	3.0882	2.2475	1.5659	3.7483	1.1248	2.3317	4.1627	8.6963	0.4872
Case 2	5	1495619	3.1045	2.2518	1.5595	3.7429	1.1275	2.3361	4.1829	8.7629	0.4826
Case 3	3.35	1495619	3.1254	2.2577	1.5524	3.7121	1.1321	2.3415	4.2076	8.8486	0.4767
Case 4	1.7	1495627	3.1556	2.2681	1.5441	3.7391	1.1392	2.3529	4.2404	8.9739	0.4689
Case 5	0.065	1495627	3.2252	2.3179	1.5569	3.7229	1.1623	2.4042	4.3192	9.2001	0.4568
Case 6 - No cranial material		1495812	3.3314	2.4100	1.5061	3.5394	1.2243	2.4953	4.4990	9.3961	0.4515
**POSTERIOR PREHENSION**	**E [GPa]**	**Elements**	**MWAM**	**MWM**	**PEofAM**	**PEofM**	**M(25%)**	**M(50%)**	**M(75%)**	**M(95%)**	**Bite Force [N]**
Case 1	6.65	1495658	3.3168	2.2854	1.4846	3.5916	1.0155	2.3675	4.5252	9.7542	0.3389
Case 2	5	1495619	3.3294	2.2913	1.4770	3.6061	1.0165	2.3739	4.5444	9.8029	0.3364
Case 3	3.35	1495627	3.3451	2.2995	1.4756	3.6298	1.0186	2.3830	4.5655	9.8669	0.3333
Case 4	1.7	1495658	3.3681	2.3127	1.4768	3.7328	1.0250	2.3990	4.5928	9.9582	0.3292
Case 5	0.065	1495207	3.4270	2.3765	1.4106	3.7534	1.0530	2.4657	4.6534	10.0920	0.3217
Case 6 - No cranial material		1495812	3.5097	2.4702	1.4517	3.5888	1.1242	2.5589	4.7747	10.1920	0.3187

GAPE 6°											
**ANTERIOR PREHENSION**	**E [GPa]**	**Elements**	**MWAM**	**MWM**	**PEofAM**	**PEofM**	**M(25%)**	**M(50%)**	**M(75%)**	**M(95%)**	**Bite Force [N]**
Case 1	6.65	1493857	3.2621	2.3664	1.5011	3.1705	1.1584	2.4414	4.4596	9.3070	0.5167
Case 2	5	1493856	3.2794	2.3720	1.4983	3.2379	1.1573	2.4488	4.4827	9.3775	0.5107
Case 3	3.35	1493856	3.3020	2.3809	1.4940	3.2601	1.1561	2.4585	4.5096	9.4704	0.5033
Case 4	1.7	1493857	3.3353	2.3973	1.4883	3.2912	1.1548	2.4762	4.5443	9.6057	0.4935
Case 5	0.065	1493857	3.4194	2.4635	1.5047	3.2503	1.2115	2.5436	4.6217	9.8644	0.4762
Case 6 - No cranial material		1493429	3.5717	2.6194	1.5881	3.4561	1.3129	2.7099	4.8566	10.1470	0.4656
**POSTERIOR PREHENSION**	**E [GPa]**	**Elements**	**MWAM**	**MWM**	**PEofAM**	**PEofM**	**M(25%)**	**M(50%)**	**M(75%)**	**M(95%)**	**Bite Force [N]**
Case 1	6.65	1493857	3.5041	2.4094	1.5422	3.5320	1.0303	2.4945	4.8783	10.4320	0.3469
Case 2	5	1493857	3.5168	2.4174	1.5394	3.5306	1.0271	2.5027	4.8977	10.4810	0.3442
Case 3	3.35	1493857	3.5334	2.4285	1.5357	3.4975	1.0242	2.5134	4.9208	10.5480	0.3407
Case 4	1.7	1493857	3.5580	2.4451	1.5306	3.5131	1.0293	2.5310	4.9496	10.6376	0.3361
Case 5	0.065	1493856	3.6249	2.5169	1.5323	3.2264	1.0899	2.5982	5.0110	10.7757	0.3277
Case 6 - No cranial material		1493431	3.7412	2.6727	1.6154	3.3461	1.2012	2.7621	5.1429	10.9200	0.3237

Supplementary Table S3. Results for anterior and posterior prehension of the ossification sequence under three alternative sequence hypothesis: L-M (Lateral to medial – preferred hypothesis for hynobiids), C-R (Caudal to Rostral – preferred hypothesis for plethodontids), R-C (Rostral to Caudal). MWAM: Mesh-Weighted Arithmetic Mean; MWM: Mesh-Weighted Median; PEofAM: Percentage Error of the Arithmetic Mean; PEofM: Percentage of Error of the Median. M: Median with subsequent quartile values. MWAM, MWM, PEofAM, PEofM are quality indicators of the mesh. M are numerical values in the quartiles of the boxplots. Bite force in Newtons (N).

ANTERIOR PREHENSION	Sequence	Elements	MWAM	MWM	PEofAM	PEofM	M(25%)	M(50%)	M(75%)	M(95%)	Bite Force [N]
Case 7 - Ossification sequence 1	L-M	1496346	3.1199	2.2503	1.4821	3.7497	1.1278	2.3347	4.1978	8.8393	0.4789
Case 8 - Ossification sequence 2	C-R	1496346	3.1617	2.2923	1.4844	3.8207	1.1421	2.3799	4.2533	8.9401	0.4700
Case 9 - Ossification sequence 3	R-C	1496307	3.1298	2.2632	1.5181	3.6600	1.1316	2.3460	4.2209	8.8655	0.4762

POSTERIOR PREHENSION	Sequence	Elements	MWAM	MWM	PEofAM	PEofM	M(25%)	M(50%)	M(75%)	M(95%)	Bite Force [N]
Case 7 - Ossification sequence 1	L-M	1496346	3.3436	2.2957	1.4212	3.5948	1.0212	2.3782	4.5584	9.8705	0.3343
Case 8 - Ossification sequence 2	C-R	1496471	3.3729	2.3352	1.4294	3.9086	1.0268	2.4265	4.5928	9.9123	0.3293
Case 9 - Ossification sequence 3	R-C	1496308	3.3495	2.3085	1.4254	3.7361	1.0210	2.3947	4.5716	9.8647	0.3335

Supplementary Table S4. Case 10 - *Dicamptodon ensatus* analyzed in the study. Muscle forces applied under surface area scaled cases (respect *S. keyserlingii* with and without fontanelle) for AME and AMI.

Case	Reference	Surface *D. ensatus* [mm^2]	Surface *S. keyserlingii* [mm^2]	AMI [MPa]	AME [MPa]
D. ensatus	*S. keyserlingii* without fontanelle	1568	194.11	0.2415	0.3010
D. ensatus	*S. keyserlingii* with fontanelle	1568	206.76	0.2267	0.2826
